# Regional distribution of excess tissue mass in ARDS lung

**DOI:** 10.1186/cc14324

**Published:** 2015-03-16

**Authors:** I Algieri, D Massari, A Colombo, G Babini, F Crimella, M Brioni, A Cammaroto, K Nikolla, C Montaruli, M Guanziroli, M Gotti, C Chiurazzi, M Amini, M Chiodi, M Cressoni, D Chiumello, L Gattinoni

**Affiliations:** 1 Università degli Studi di Milano, Milan, Italy; 2 Fondazione IRCCS, 'Ospedale Maggiore Policlinico Mangiagalli Regina Elena' di Milano, Milan, Italy

## Introduction

ARDS is characterized by edema diffuse to all lung fields. Distribution of excess tissue mass had been studied with CT scan in a few patients on a single slice, comparing with data obtained in healthy controls.

## Methods

ARDS patients underwent CT scan imaging during their ICU stay at 45 cmH_2_O end-inspiratory pressure. After hospital discharge, patients underwent a follow-up CT scan performed at end inspiration. Each lung was divided into three sections along the apex-base axis and into three sections along the sternum-vertebral axis (nine regions per lung). Excess tissue mass in each lung region was defined as the difference in lung tissue (grams) between the CT scan performed during ARDS course and the follow-up CT scan. Results are presented as mean ± SD.

## Results

**Figure 1:**
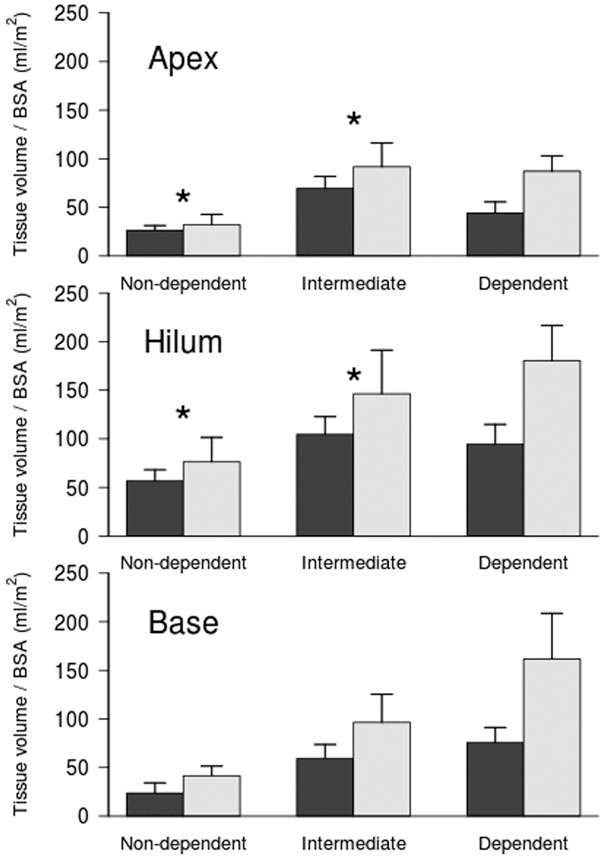


We studied eight ARDS patients (55 ± 18 years) with a BMI of 27 ± 6 kg/m^2^. At ICU admission, patients had the following clinical parameters: PaO_2_/FiO_2_ 106 ± 33 with PEEP 15 ± 5 cmH_2_O; PaCO_2_ 43 ± 10 mmHg; pH 7.35 ± 0.05. The average increase in lung weight during ARDS compared with follow-up CT scan was 68 ± 40% (680 ± 320 g). Figure 1 presents the tissue volume during ARDS (white bars) and after ARDS resolution (black bars) and compares the ratio between the two (*P < 0.01 vs. dependent region).

## Conclusion

The excess tissue mass was not different between apex, hilum and base, but was increased in the dependent lung regions at apex and hilum, being uniformly distributed at the lung base.

